# Bone marrow mesenchymal stem cells protected post-infarcted myocardium against arrhythmias *via* reversing potassium channels remodelling

**DOI:** 10.1111/jcmm.12287

**Published:** 2014-04-30

**Authors:** Benzhi Cai, Gang Wang, Nan Chen, Yanju Liu, Kun Yin, Chunping Ning, Xingda Li, Fan Yang, Ning Wang, Yang Wang, Zhenwei Pan, Yanjie Lu

**Affiliations:** a Department of Pharmacology, State-Province Key Laboratories of Biomedicine-Pharmaceutics of China, Key Laboratory of Cardiovascular Research, Ministry of Education, Harbin Medical UniversityHarbin, Heilongjiang Province, China; b China-Russia Medicine Research Center, Harbin Medical UniversityHarbin, Heilongjiang Province, China; c Department of Ultrasound, The Affiliated Second Hospital of Harbin Medical UniversityHarbin, Heilongjiang Province, China

**Keywords:** bone marrow mesenchymal stem cells, myocardial infarction, arrhythmias, potassium channels, calcineurin

## Abstract

Bone marrow mesenchymal stem cells (BMSCs) emerge as a promising approach for treating heart diseases. However, the effects of BMSCs-based therapy on cardiac electrophysiology disorders after myocardial infarction were largely unclear. This study was aimed to investigate whether BMSCs transplantation prevents cardiac arrhythmias and reverses potassium channels remodelling in post-infarcted hearts. Myocardial infarction was established in male SD rats, and BMSCs were then intramyocardially transplanted into the infarcted hearts after 3 days. Cardiac electrophysiological properties in the border zone were evaluated by western blotting and whole-cell patch clamp technique after 2 weeks. We found that BMSCs transplantation ameliorated the increased heart weight index and the impaired LV function. The survival of infarcted rats was also improved after BMSCs transplantation. Importantly, electrical stimulation-induced arrhythmias were less observed in BMSCs-transplanted infarcted rats compared with rats without BMSCs treatment. Furthermore, BMSCs transplantation effectively inhibited the prolongation of action potential duration and the reduction of transient and sustained outward potassium currents in ventricular myocytes in post-infarcted rats. Consistently, BMSCs-transplanted infarcted hearts exhibited the increased expression of K_V_4.2, K_V_4.3, K_V_1.5 and K_V_2.1 proteins when compared to infarcted hearts. Moreover, intracellular free calcium level, calcineurin and nuclear NFATc3 protein expression were shown to be increased in infarcted hearts, which was inhibited by BMSCs transplantation. Collectively, BMSCs transplantation prevented ventricular arrhythmias by reversing cardiac potassium channels remodelling in post-infarcted hearts.

## Introduction

Cardiovascular diseases remain a leading cause of death all over the world. Recently, stem cells have emerged as a promising resource for the treatment of heart diseases [1]. As one kind of adult stem cells, bone marrow mesenchymal stem cells (BMSCs) are capable to release cytokines such as VEGF, IGF-1 and bFGF which can protect heart against ischaemic injury and prevent cardiac fibrosis [1–3]. Accordingly, BMSCs become an attractive stem cell candidate for cardiovascular repair [4,5]. Experimental studies have confirmed that the implantation of culture-expanded BMSCs reduced myocardial scar and infarct size, improved heart function and increased vascular density [2,6–8]. Therapeutic effects of BMSCs have also been clarified on diabetic cardiomyopathy [9,10], dilated cardiomyopathy [2], acute myocarditis [11] and heart failure [12,13]. Recently, a series of clinical trials reported that BMSCs transplantation had the high feasibility to treat heart disease in patients [14,15]. Despite its strong ability to heal damaged hearts, the influence or role of BMSCs on cardiac electrophysiological properties remains unclear.

It is well known that potassium channels remodelling provides electrophysiological substrate for ventricular arrhythmias or sudden cardiac death in heart disease [16,17]. Transient outward potassium currents (I_to_) and sustained outward potassium channels (I_KSUS_) were considerably reduced under some pathological conditions, which caused the prolongation of action potential duration (APD) and delayed repolarization of action potential in cardiomyocytes [16,18]. Molecular investigations demonstrated that the down-regulation of potassium channel subunits such as K_V_4.2, K_V_4.3 and K_V_2.1 contributed to the decrease in outward potassium currents and subsequent ventricular arrhythmias in heart infarction [18]. Accordingly, it is considered as an important strategy to prevent arrhythmias after heart infarction by reversing potassium channels remodelling.

Nevertheless, there is, so far, no information about the effects of transplanted BMSCs on potassium channel remodelling after heart infarction. The present study was aimed to investigate whether BMSCs transplantation may improve potassium channel remodelling in infarcted rats and explore its potential mechanisms.

## Materials and methods

### Bone marrow mesenchymal stem cells

Bone marrow mesenchymal stem cells were isolated and cultured as described in previous report [19]. In brief, bone marrow cells from femurs and tibias were flushed into a beaker and then transferred into culture flasks with Basal Medium for Mesenchymal Stem Cells (Stem Cell Technologies Inc., Vancouver, BC, Canada) supplemented with 20% Mesenchymal Stem Cell Stimulatory Supplements (Stem Cell Technologies Inc.) and penicillin (100 U/ml)/streptomycin (100 U/ml) at 37°C in humid air with 5% CO_2_. After cultured for 3 days, the adherent layer was washed with the fresh medium and then cultured continuously. The cultured cells were passaged at 1:2 dilution after reaching 80% confluence by 0.25% Trypsin (Sigma-Aldrich, St. Louis, MO, USA) treatment. All experiments were performed with the cells of the third passage.

### Myocardial infarction and BMSCs transplantation

Male adult SD rats (240–300 g) were purchased from the Experimental Animal Center of Harbin Medical University (Harbin, China) and were kept in plastic cages under conditions of controlled temperature (18–21°C) and humidity (55 ± 5%) with a 12/12 hrs light/dark cycle. All procedures on animals are in accordance with the guidelines of the Animal Ethics Committee of Harbin Medical University. Male SD rats were divided into three groups: sham operation (Control group; 20 rats); myocardial infarction (MI group; 20 rats); heart infarction with BMSCs transplantation (BMSCs+MI group; 20 rats). Male SD rats were anaesthetized by IP injection of pentobarbital 30 mg/kg, and then ECG was recorded by a standard recorder. The method to establish myocardial infarction in rats by left anterior descending coronary artery ligation was described previously [20,21]. In brief, rat hearts were exposed after chest was opened. The left anterior descending coronary artery was occluded with a 6-O braided silk suture about 2 mm below its origin. Successful occlusion was confirmed by pallor of the anterior wall of the LV and ST segment elevation. The ligature was put in place but was not tied in sham-operated animals. Three days later, the third passage BMSCs (1 × 10^6^) were intramyocardially injected into the infarcted zone of hearts at four points. The other two groups of rats underwent the sham operation and were injected with the same volume of PBS at the same four sites. The rat mortality after coronary artery ligation was continuously recorded after heart operation. The rats used for haematoxylin and eosin staining, Masson staining and western blot in three groups were killed in the same day. The rats that were prepared for calcium transient and patch-clamp recordings underwent the surgery separately.

### Haematoxylin and eosin and Masson's trichrome staining

Harvested myocardium from the LV of rats was immediately placed in pre-cooled 4% paraformaldehyde and further fixed overnight at 4°C. The fixed tissues were then embedded in paraffin for histological studies. The paraffin-embedded tissues were processed for sectioning. Tissue sections were then stained with haematoxylin and eosin and Masson's trichrome for the assessment of myocardial structure and fibrosis. Images were visualized under an optical microscope at ×200 magnification. Three rats in each group were used for haematoxylin and eosin and Masson staining.

### Echocardiography

Echocardiography was performed on the rats under anaesthesia 2 weeks after BMSCs transplantation. Echocardiograms were performed by using a HP Sonos 2500 echocardiographic system (Hewlett–Packard, New Orleans, LA, USA) with a 10 MHz imaging linear scan probe transducer. The LV end-systolic (LVIDd) and end-diastolic (LVIDs) dimensions were measured on two-dimensionally guided M-mode tracings through the anterior and posterior walls of the LV. Ejection fraction (EF) was calculated from the M-mode LV dimensions as described previously [22]. Parameters for the calculation of heart function were measured from three consecutive systole-diastole cycles, were performed by an experienced technician and were analysed by an echocardiography expert and a cardiologist.

### Measurement of intracellular free calcium

Rat ventricular myocytes were loaded with Fluo-3/AM at 37°C for 45 min. and then washed with Tyrode's solution three times. After loading with Fluo-3/AM, the glass coverslips with isolated ventricular myocytes were transferred into a recording chamber and superfused with Tyrode's solution. Fluorescent changes of Fluo-3/AM-loaded cells were detected using laser scanning Confocal microscope (Olympus FV-300, Tokyo, Japan). The fluorescent intensities were both recorded before (F0) and after (FI) the cells were subjected to 2 mV electrical stimuli at 0.5 HZ. Qualitative changes of intracellular Ca^2+^ level were inferred from the ratio of FI/F0. Three rats in each group were chosen for subsequent calcium transient recording.

### Isolation of ventricular myocytes

The method to isolate ventricular myocytes of rats was described previously [21,23]. In detail, the hearts from seven rats in each group were mounted on a modified Langendorff perfusion system. The heart was perfused with Ca^2+^-containing Tyrode's solution (in mM: NaCl 126, KCl 5.4, MgCl_2_ 1, CaCl_2_ 1.8, NaH_2_PO_4_ 0.33, glucose 10, and Hepes 10, pH 7.4, with NaOH), then was transferred to Ca^2+^-free Tyrode's solution at 37°C and continually perfused with Ca^2+^-free Tyrode's solution containing collagenase (type II) and 1% bovine serum albumin. The ventricular myocytes from the border zone of infarcted hearts or from the same area of sham group rats were excised from the softened hearts, minced and placed in a KB medium (in mM: glutamic acid 70, taurine 15, KCl 30, KH_2_PO_4_ 10, MgCl_2_ 0.5, EGTA 0.5, Hepes 10, and glucose 10, pH 7.4, with KOH) at 4°C for 1 hr before recordings.

### Patch clamping

The whole-cell patch-clamp techniques were used to record potassium currents in the voltage-clamp mode, and action potential in the current-clamp mode using an Axopatch 200B amplifier (Axon Instruments, Foster City, CA, USA) [24]. Borosilicate glass electrodes with tip resistances from 2 to 4 MΩ were connected to a headstage (Axon Instruments). The pipette potential was zeroed before touching cardiomyocytes membrane. Voltage pulses were generated by a 12-bit digital-to-analog converter controlled by pClamp 9.0 software (Axon Instruments). Junction potentials between bath and pipette solution averaged <10 mV and were corrected for action potential only. After seal formation, cellular membrane was ruptured by gentle suction to establish the whole-cell configuration. The capacitance and series resistance were compensated and potassium currents were expressed as current density (pA/pF) by normalizing the current to its capacitance for every cardiomyocytes. Myocytes with significant leak currents were rejected. Tetrodotoxin (30 nM) and verapamil (1 μM) were added to the Tyrode's solution to block the fast sodium and calcium inward currents in isolated cardiomyocytes.

### Western blot

The protein samples were extracted from the LV of rats with the procedures as previously described [25]. The protein concentration was determined using the BCA method as recommended by the manufacturer. After boiled for 5 min., the protein samples were fractionated by SDS-PAGE (10–15% polyacrylamide gels) and transferred to PVDF membrane (Millipore, Bedford, MA, USA). The samples were blocked with milk powder for 1 hr at room temperature and then incubated with primary antibodies K_V_1.4, K_V_1.5, K_V_2.1, K_V_4.2, K_V_4.3 (Alomone Labs, Jerusalem, Israel) as well as calcineurin (Santa Cruz Biotechnology Inc., Santa Cruz, CA USA) and NFATc3 (Santa Cruz Biotechnology Inc.) at 4°C overnight. After washing, the membranes were incubated with a secondary antibody for 1 hr at room temperature. Western blot bands were quantified using Odyssey v1.2 software by measuring the band intensity (area × OD) for each group and normalizing to GAPDH as an internal control.

### Statistical analysis

All experimental data were presented as the mean ± SEM. anova or *t*-test was used to compare mean values using SPSS 13.0 software (SPSS Inc, Chicago, IL, USA). Values of *P* < 0.05 were considered statistically significant.

## Results

### BMSCs transplantation improved heart function of infarcted myocardium

**Fig. 1:**
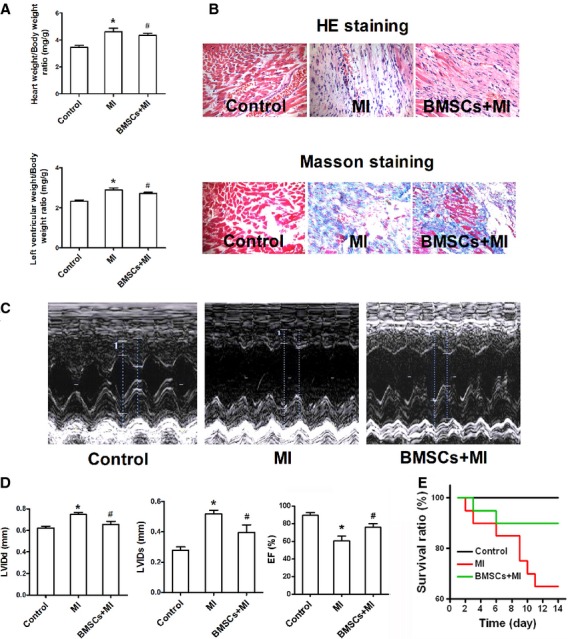
Effects of bone marrow mesenchymal stem cells (BMSCs) transplantation on heart structure and function in infarcted rats. (**A**) Heart weight/bw ratio and LV weight/bw ratio were measured in control rats, infracted rats and BMSCs-transplanted infarcted rats (*n* = 13). (**B**) Haematoxylin and eosin and Masson trichrome's staining of LV sections from control rats, infracted rats and BMSCs-transplanted infarcted rats. (**C**) Representative traces of echocardiography performed under control rats as well as in infarcted rats with and without BMSCs transplantation. (**D**) Measurement of LVIDd, LVIDs and ejection fraction among three groups (*n* = 8). Echocardiography measurement showed a significant difference in these parameters between infarcted hearts and BMSCs-treated infarcted hearts. (**E**) Percentages of survival in control rats, infarcted rats and BMSCs-treated infarcted rats (*n* = 20). *P < 0.05 *versus* control, ^#^*P* < 0.05 *versus* MI.

To confirm that BMSCs play a protective role in post-infarcted hearts *in vivo*, culture-expanded BMSCs were transplanted into LV myocardium *via* intramyocardial delivery 3 days after coronary artery ligation. Two weeks later, the infarcted rats developed an increase in heart/bw ratio as well as LV weight ratio (Fig. 1A). These remodelling phenotypes were attenuated in infarcted rats with BMSCs transplantation. Haematoxylin and eosin and Masson's trichrome staining demonstrated that BMSCs transplantation ameliorated the destroyed heart structure and augmented cardiac fibrosis in infarcted myocardium (Fig. 1B). In agreement, echocardiography examinations showed that BMSCs transplantation improved heart function in infarcted rats (Fig. 1C and D). The engrafted BMSCs reduced the LVIDd, LVIDs and improved the EF in infarcted hearts. In addition, we also observed that the rats with BMSCs transplantation exhibited an improvement of survival of infarcted rats within 2 weeks after BMSCs transplantation (Fig. 1E).

### BMSCs decreased the susceptibility of infarcted hearts to arrhythmias

**Fig. 2:**
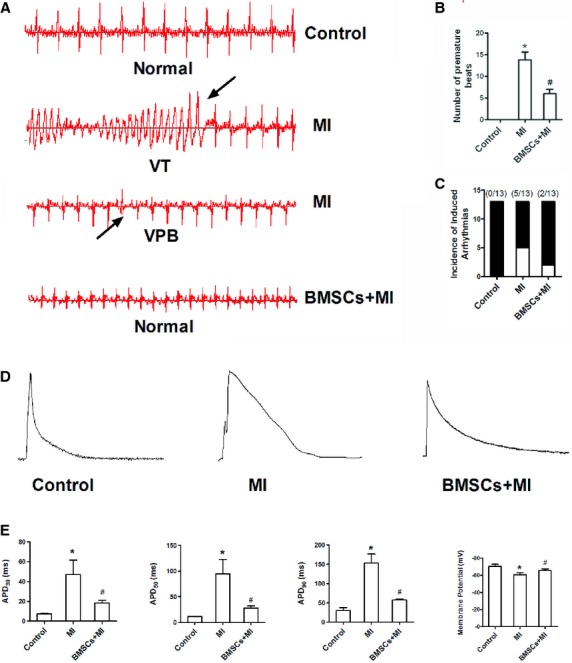
Influences of bone marrow mesenchymal stem cells (BMSCs) transplantation on ventricular arrhythmias and action potential in infarcted rats. (**A**) Representative surface 3-lead ECG was recorded in three groups of rats. (**B**) The number of ventricular arrhythmias after the induction of electrical stimuli was increased in infarcted rats, which can be attenuated after BMSCs transplantation. (**C**) BMSCs transplantation decreased the incidence of arrhythmias in heart with myocardial infarction. (**D**) Action potentials recorded in LV myocytes of border zone from control rats, infarcted rats and BMSCs-treated infarcted rats. (**E**) Action potential duration at 30, 50 and 90% of full repolarization (APD30, APD50 and APD90) of LV myocytes obtained from three groups of rats (*P* < 0.05). Comparison of resting membrane potentials recorded in ventricular myocytes from three groups of rats. *P < 0.05 *versus* control, ^#^*P* < 0.05 *versus* MI.

Sustained infarction leads to cardiac electrophysiological remodelling and then provides substrate for ventricular arrhythmias or sudden death after pathological stimuli. We then observed whether BMSCs exerted preventive effects on electrical-induced arrhythmias of infarcted rats (Fig. 2A). As depicted in Figure 2B and C, the number and incidence of ventricular premature beat or arrhythmias were higher in infarcted hearts, which was decreased in infarcted rats with BMSCs transplantation. Evidently, these results suggest that BMSCs transplantation may decrease arrhythmic risk of infarcted hearts in response to electrical stimuli.

### BMSCs reversed the prolongation of action potential duration in infarcted hearts

Arrhythmias in infarcted hearts were associated with abnormal repolarization of action potential of ventricular myocytes [16]. Thus, we employed patch champ technique to record action potential in border-zone ventricular myocytes. Figure 2D demonstrated the representative traces of action potential of ventricular myocytes from control group, infarcted group and BMSCs-transplanted infarcted group. Action potential duration at 30% repolarization (APD30, 7.3 ± 0.6, 47.3 ± 14.4 and 18.4 ± 2.8 ms respectively), 50% repolarization (APD50, 11.2 ± 0.8, 94.7 ± 28.0 and 27.9 ± 4.1 ms respectively) and 90% repolarization (APD90, 30.2 ± 7.4, 152.9 ± 23.8 and 57.3 ± 2.6 ms respectively) was prolonged in rats subjected to myocardial infarction (Fig. 2E), which was partially reversed in BMSCs-transplanted infarcted hearts. In addition, BMSCs transplantation also inhibited the shift of resting membrane potential towards the depolarizing direction in infarcted hearts (−70.3 ± 2.6, −54.6 ± 1.9 and −65.6 ± 1.7 mV respectively). However, there were no significant differences in AP amplitude and maximum upstroke velocity (Vmax) between infarcted rats and BMSCs-transplanted infarcted rats.

### BMSCs attenuated the reduction of outward potassium currents in infarcted hearts

**Fig. 3:**
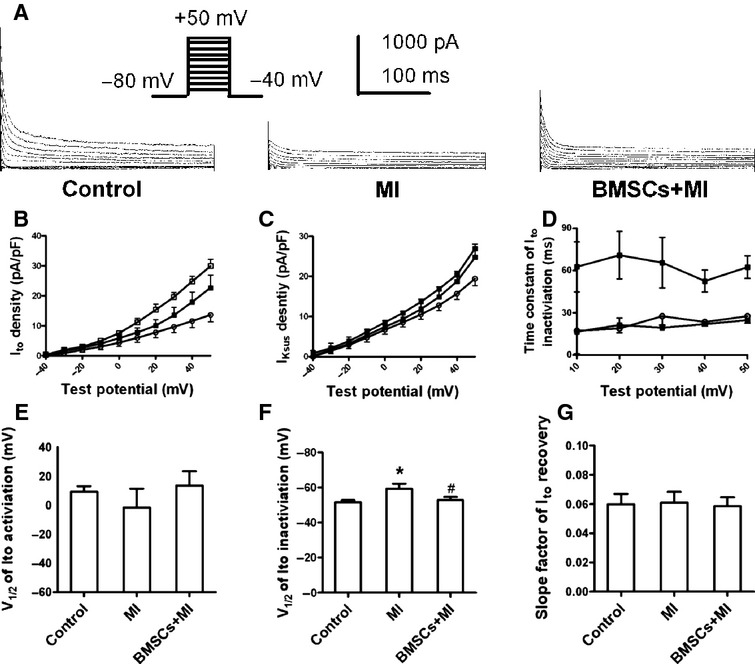
Bone marrow mesenchymal stem cells (BMSCs) affected transient and sustained outward potassium currents in infarcted rats. (**A**) Representative traces of Ito recorded in LV myocytes of infarcted hearts with and without BMSCs transplantation (**B**). The average current–voltage relationship curve of Ito recorded in cardiac myocytes of three groups of rats. Ito was quantified by subtracting the currents at the end of the voltage pulse (300 ms) from the peak currents. (**C**) The current–voltage relationship curve of I_K_sus in LV myocytes from three groups. (**D**) The inactivation time constants (τ) of Ito were estimated at test potentials ranging from +10 mV to +50 mV by the exponential fitting of the current decay. (**E**) V1/2 of the steady-state activation curve of Ito was calculated among three groups. (**F**) The comparison of V1/2 of the steady-state inactivation curve of Ito among three groups. (**G**) The slope factor of the recovery of Ito from inactivation was not altered among three groups. *P < 0.05 *versus* control, ^#^*P* < 0.05 *versus* MI.

Potassium currents play a key role in the repolarization of action potential in cardiac myocytes [26]. Accordingly, we further observed the effects of BMSCs transplantation on potassium currents in infarcted hearts. Ito was recorded with 300 ms depolarizing pulses between −40 and +50 mV from a HP of −80 mV. As shown in Figure 3A, Ito was reduced in LV myocytes isolated from the border zone of infarcted hearts, which was partially ameliorated in the myocytes from the border zone of BMSCs-transplanted infarcted hearts. Figure 3B displayed the current–voltage (I–V) relationship curve of Ito in control rats, infarcted rats and BMSCs-treated infarcted rats. The current density of transient Ito at +50 mV was 29.9 ± 2.3 pA/pF in control rats, 13.6 ± 2.3 pA/pF in infarcted rats and 22.7 ± 4.3 pA/pF in BMSCs-transplanted infarcted rats respectively (*n* = 8, 12 and 11 respectively, *P* < 0.05). Also, sustained outward potassium currents (I_KSUS_) contribute to the repolarization of action potential. As displayed in Figure 3C, the current density of I_KSUS_ at +50 mV was markedly decreased in the infarcted rats from 27.0 ± 4.5 pA/pF to 19.5 ± 1.5 pA/pF and recovered to 24.7 ± 2.8 pA/pF in BMSCs-transplanted infarcted rats. Compared with control cells, the time constant of the decay of Ito traces was slowed in ventricular myocytes of border zone of infarcted rats, which was attenuated in cardiomyocytes from BMSCs-transplanted infarcted rats (18.9 ± 3.0 ms in control group, 70.8 ± 16.8 ms in MI group and 21.3 ± 4.9 ms in BMSCs group, respectively, *P* < 0.05, at +20 mV; Fig. 3D).

The kinetic properties of Ito in three groups were further analysed. There was no significant difference of V1/2 of the voltage-dependent Ito activation among three groups (9.4 ± 3.7, −2.3 ± 13.1 and 13.5 ± 9.9 mV in control, MI and BMSCs group, respectively, *P* > 0.05; Fig. 3E). The voltage-dependent inactivation of Ito was obtained with 400-ms pre-pulses between −100 and +40 mV from a HP of −80 mV, followed by a 300-ms test pulse to +60 mV. V1/2 of the voltage-dependent Ito inactivation was altered in infarcted rats, compared with control rats. The changes of V1/2 of Ito inactivation were reversed in BMSCs-transplanted rats (−51.6 ± 1.1 mV, −59.1 ± 3.0 mV and −52.7 ± 1.7 mV in control, MI and BMSCs group, respectively, *P* < 0.05; Fig. 3F). The recovery of Ito from inactivation was assessed with the use of paired pulses (P1, P2) of 300-ms duration to +50 mV from a HP of −80 mV with varying interpulse intervals (20 ms increment) at 0.2 Hz. No significant differences were observed among three groups (0.059 ± 0.007, 0.061 ± 0.007 and 0.058 ± 0.006 in control, MI and BMSCs group, respectively, *P* > 0.05; Fig. 3G).

### BMSCs reversed cardiac potassium channels remodelling in infarcted hearts

**Fig. 4:**
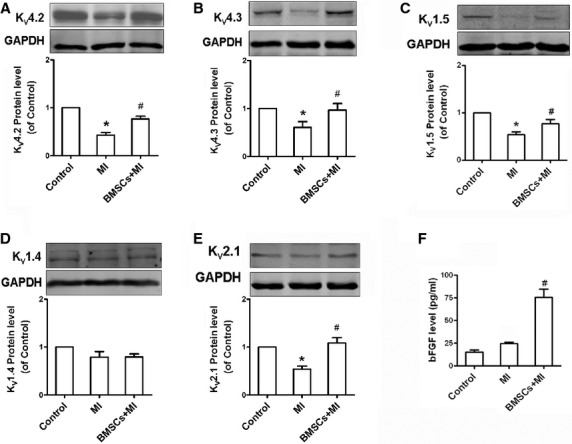
Bone marrow mesenchymal stem cells (BMSCs) transplantation reversed the changes of K_V_4.2, K_V_4.3, K_V_1.5 and K_V_2.1 proteins in infarcted rats. Western blotting analysis of K_V_4.2 (**A**), K_V_4.3 (**B**), K_V_1.5 (**C**), K_V_1.4 (**D**) and K_V_2.1 (**E**) proteins in LV myocytes of the border zone from control, infarcted and BMSCs-treated infarcted rats. (**F**) The bFGF level was elevated in infarcted myocardium with BMSCs transplantation. *P < 0.05 *versus* control, ^#^*P* < 0.05 *versus* MI.

We further investigate whether the subunits of cardiac potassium channels are altered in infarcted and BMSCs-transplanted infracted hearts. As shown in Figure 4A and B, the myocardium from infarcted rats demonstrated a reduction in K_V_4.2 and K_V_4.3, compared with control hearts. Bone marrow mesenchymal stem cells-transplanted infracted rats exhibited an augmented expression of K_V_4.2 and K_V_4.3 subunits in hearts compared with infarcted rats. In addition, the expression of K_V_1.4, K_V_1.5 and K_V_2.1 proteins which form I_KSUS_ in rat ventricular myocytes was further observed in three groups. Figure 4C and E demonstrated that K_V_1.5 and K_V_2.1 but not K_V_1.4 subunits were reduced in infarcted hearts compared with control hearts. BMSCs transplantation can reverse the reduction in K_V_1.5 and K_V_2.1 protein levels in hearts of infarcted rats. BMSCs transplantation also inhibited the down-regulation of Kir2.1 in infracted heart (Fig. S1). These suggest that BMSCs treatment leads to the reversal of cardiac potassium channel remodelling following myocardial infarction. Our previous study has shown that bFGF can increase I_to_ by up-regulating its mRNA expression [19]. Consistently, the level of bFGF was observed higher in BMSCs-treated hearts than MI hearts (Fig. 4F).

### BMSCs transplantation altered intracellular calcium transient in infarcted hearts

**Fig. 5:**
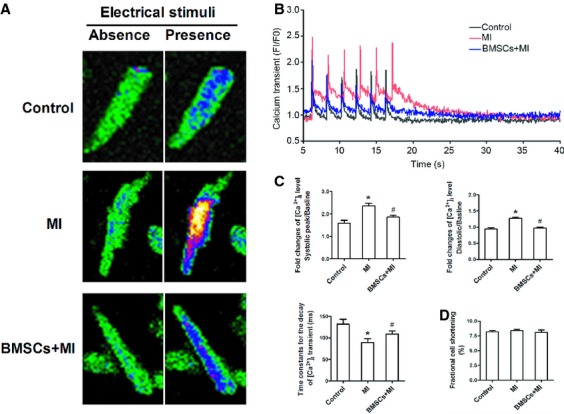
Effects of bone marrow mesenchymal stem cells (BMSCs) transplantation on intracellular calcium homoeostasis of LV myocytes in infarcted hearts. (**A**) Representative image of intracellular free calcium transients from LV cells from three groups. (**B** and **C**) The change in systolic and diastolic intracellular free calcium relative to baseline is higher in infarcted hearts than in BMSCs-transplanted infarcted hearts, and the decay time of calcium transient in the myocytes of the border zone from three groups. (**D**) The comparison of fractional cell shortening relative to baseline of LV cells from three groups. *P < 0.05 *versus* control, ^#^*P* < 0.05 *versus* MI.

Cardiac potassium channels remodelling is associated with the overload of intracellular Ca^2+^ concentration [27], which then regulates potassium channel genes expression *via* calcineurin/NFATc3 signalling pathway [28]. We therefore investigated whether disturbed intracellular calcium homoeostasis was restored in infarcted heart with BMSCs transplantation. As shown in Figure 5, LV myocytes from the border zone of infarcted hearts exhibited a significant increase in systolic and diastolic intracellular Ca^2+^ transient relative to baseline as compared with the cells from control rats, and this alteration can be attenuated in ventricular myocytes from infarcted rats following BMSCs transplantation (Fig. 5B and C). In addition, the decay time of calcium transient was also considerably prolonged in infarcted myocardium and was shorter in cardiomyocytes from infracted hearts with BMSCs transplantation (Fig. 5C). These data suggest that the alteration of intracellular calcium transient was involved in the preventive effects of BMSCs on potassium channels remodelling after myocardial infarction. We also compared fractional cell shortening among all three groups, and the results showed that cell shortening of ventricular myocytes was not altered in infarcted myocardium and also in infarcted myocardium with BMSCs transplantation (Fig. 5D).

### BMSCs transplantation affected calcineurin/NFATc3 signalling pathway

**Fig. 6:**
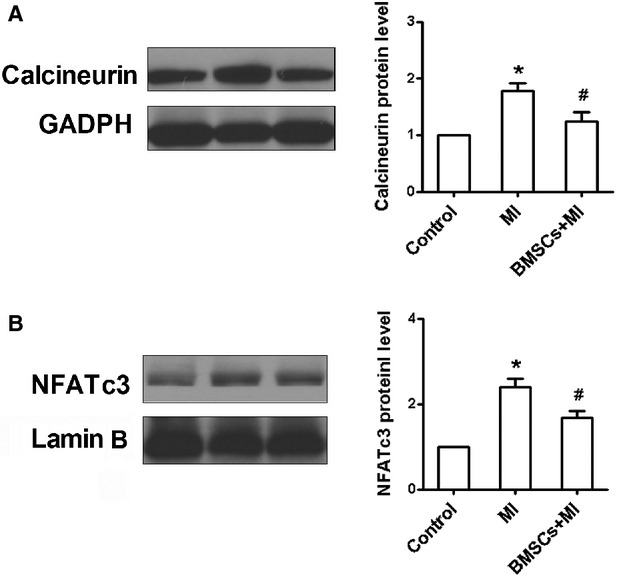
The expression of calcineurin protein measured in infarcted hearts and bone marrow mesenchymal stem cells (BMSCs)-transplanted hearts. (**A**) The rat with myocardium infarction showed the increased calcineurin expression, which was partially reserved by BMSCs transplantation. (**B**) The increased expression of NFATc3 in the nuclear of ischaemic heart cells was attenuated after BMSCs treatment. *P < 0.05 *versus* control, ^#^*P* < 0.05 *versus* MI.

Previous studies uncovered that the activation of calcineurin/NFATc3 signalling pathway might contribute to potassium channel pattern in heart diseases [28,29]. Conversely, targeted disruption of NFATc3 resulted in an intrinsic loss of calcineurin-mediated potassium channel remodelling. So, we further study whether calcineurin/NFATc3 signalling pathway accompanies the observed BMSCs-induced prevention of cardiac potassium channel remodelling. As showed in Figure 6A, an increase in calcineurin protein was found in rats with heart infarction, which was decreased in the rats with BMSCs transplantation. Furthermore, the expression of nuclear NFATc3 protein was also reduced in BMSCs-treated infarcted myocardium (Fig. 6B). These suggest that BMSCs transplantation attenuates myocardial infarction-induced potassium channels remodelling possibly through blocking calcineurin/NFATc3 signalling pathway, which partially contributes to the protective effects of BMSCs on post-infarcted arrhythmias.

## Discussion

Firstly, we showed that BMSCs transplantation prevented potassium channel remodelling after heart infarction and decreased ventricular arrhythmia susceptibility in infarcted myocardium. The inhibition of Ca^2+^/Calcineurin/NFatc3 signalling pathway may partially explain the decrease in arrhythmia susceptibility. These findings provide insights into the biological actions of BMSCs on hearts.

Bone marrow mesenchymal stem cells transplantation has attracted considerable attention as a new approach of cardiac repair [30]. A large body of evidence indicated that BMSCs exerted protective effects on myocardial infarction [7], heart failure [13,30] and cardiac fibrosis [12]. Engrafted BMSCs may prevent local cardiomyocytes apoptosis, and reverse extracellular matrix remodelling in heart failure following myocardial infarction, without influences on cardiac enzyme release and systemic inflammatory markers [13]. As a common complication of heart diseases, cardiac arrhythmias are a major risk factor for the mortality of those patients. The present study was therefore aimed to clarify whether BMSCs transplantation improves cardiac electrophysiological remodelling after heart infarction.

In agreement with previous reports, our study also confirmed that heart structure and function were significantly improved in infarcted rats after BMSCs transplantation [31]. Bone marrow mesenchymal stem cells have been shown to increase cellular viability and decrease collagen secretion of cardiac fibroblasts *via* the stimulation of MMP-2/MMP-9 activities [12]. We also found that BMSCs had an inhibitory effect on cardiac fibrosis. Furthermore, we uncovered that the survival of infarcted rats was higher in BMSCs-transplanted rats than non-transplanted rats, and electrical-induced arrhythmias were also seldom observed in infarcted hearts with BMSCs transplantation. It implies that BMSCs play a preventive role in post-infarcted ventricular arrhythmias. Though the concern on the proarrhythmic potential of BMSCs engraftment was raised, the risk of ventricular arrhythmias after MI was shown decreased in rats with BMSCs injection [32,33]. Indeed, recent study implied that the engrafted BMSCs were able to couple with host cardiomyocytes with the increasing expression of cardiomyocytes-specific markers [33,34]. It was reported that BMSCs produced preventive effects on heart rhythm disorders by reversing connexin43 down-regulation and up-regulating transient outward potassium currents [19,32,34]. Consistently, our results confirmed that BMSCs transplantation may decrease the susceptibility of infarcted heart to arrhythmias, which provides novel evidence for the prevention of heart diseases using BMSCs. It was shown that that the prolongation of APD may induce early afterdepolarization in ventricular myocytes and ventricular arrhythmias [16]. In this study, electrophysiological recordings also showed APD was markedly prolonged in the heart of infarcted rats, but this alteration was reversed in infarcted hearts after BMSCs transplantation. Prolongation of APD usually occurs as a result of the decrease in outward potassium currents in ventricular myocytes of infarcted hearts [18]. We therefore investigated the difference of Ito and I_Ksus_'currents of ventricular myocytes among three groups. The results showed that Ito and I_Ksus_ were significantly inhibited in border-zone cardiomyocytes after heart infarction. Nevertheless, BMSCs transplantation can rescue the reduction in Ito and I_Ksus_ in border-zone cardiomyocytes. Previous studies uncovered that some subunits of cardiac potassium channels were down-regulated in ischaemic or hypertrophic heart diseases, which causes the decrease in outward potassium currents and subsequent ventricular arrhythmias in heart infarction [35]. So, the effects of BMSCs transplantation on these potassium channel subunits were further studied. We found that BMSCs-transplanted hearts exhibited increased expression of K_V_4.2, K_V_4.3, K_V_1.5, K_V_2.1 and Kir2.1 proteins in ventricular myocytes when compared to with non-transplanted infracted rats. The down-regulation of K_V_4.2 and K_V_4.3 caused the reduction in Ito in infarcted myocardium. In addition, we observed a negative shift of Ito inactivation which will result in the rapid closure of potassium channel and the decrease in potassium currents, also contributing to the reduction in I_to_ in infarcted myocardium. So, it means that the changes of Ito inactivation and potassium channel down-regulation both will lead to the decrease in Ito.

Intracellular free calcium level ([Ca^2+^]i) is an important messenger and involved in many pathological conditions. The overload of intracellular Ca^2+^ level facilitates cardiac structural and electrical remodelling by activating downstream signalling pathways [36]. Previous studies showed that intracellular Ca^2+^ overload altered the expression of potassium channel genes in cardiac cells *via* regulating Ca^2+^/calcineurin/NFATcs pathway [37]. Therefore, targeting intracellular Ca^2+^ level is an important approach to prevent cardiac electrophysiological remodelling in response to pathological stimuli. In this study, infarcted myocardium exhibited a significant elevation of intracellular Ca^2+^ transient. Intracellular calcium level is determined by L-type calcium currents and SR uptake and release. It was reported that L-type calcium current was decreased or not changed in infarcted myocardium. It indicates that L-type calcium channel in MI will not contribute to the increase in intracellular calcium. Meanwhile, we observed an increase in both systolic and diastolic calcium transient, suggesting the dysfunction of SR in calcium uptake and release. Therefore, we speculated that increased intracellular calcium resulted from the SR dysfunction, but not L-type calcium channel. BMSCs-transplanted infarcted hearts did not show an augmented Ca^2+^ transient in response to electrical stimuli. Meanwhile, the expression of calcineurin and nuclear NFATc3 proteins was also inhibited in BMSCs-treated infarcted hearts. These suggest that BMSCs may reverse potassium channels remodelling *via* blocking Ca^2+^/calcineurin/NFATc3 signalling pathway after myocardial infarction. Previous study also showed that increased concentration of free Ca^2+^ shifted Ito inactivation in a negative direction [38]. Thus, we speculated that increased intracellular Ca^2+^ led to the negative shift of Ito inactivation.

In this study, we intramyocardially transplanted 1 × 10^6^ BMSCs into rat hearts based on the previous studies with some modifications [2,11]. The complete mechanism underlying preventive effects of BMSCs on cardiac potassium channel remodelling is complex. Our previous research has reported that BMSCs impact electrophysiological properties of ventricular myocytes *via* secreting soluble factors such as bFGF [19]. Based on this, it is supposed that BMSCs transplantation improved cardiac electrical remodelling *via* its paracrine actions on potassium channels of cardiomyocytes or *via* paracrine-induced cardioprotection [31]. Of course, BMSCs-based therapy still remains at the exploring stage and requires the long-term evaluation of potential whole effects.

## Conclusion

In summary, here we found that intramyocardial transplantation of BMSCs could reverse cardiac electrical remodelling and prevent ventricular arrhythmias in infarcted hearts, which provide us new insights into understanding the biological actions of BMSCs on heart diseases.

## Supporting information

**Figure S1**. Effects of BMSCs transplantation on Kir2.1 expression in infracted hearts.
