# Supplementary material for: Bone marrow mesenchymal stem cells protected post-infarcted myocardium against arrhythmias via reversing potassium channels remodelling
Source: J Cell Mol Med. 2014 Apr 30;18(7):1407–16. doi: 10.1111/jcmm.12287 (PMC4124024; doi:10.1111/jcmm.12287)
Supplement: Supplementary file 1 — Figure S1. Effects of BMSCs transplantation on Kir2.1 expression in infracted hearts. [file jcmm0018-1407-SD1.doc]

Supplementary Figure 1.


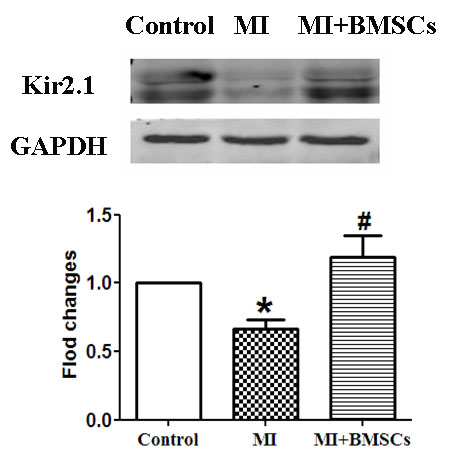


Supplementary Figure 1. Effects of BMSCs transplantation on Kir2.1 expression in infracted hearts. The expression of Kir2.1 which forms I_KI_ was detected in infarcted heart with or without BMSCs treatment. The results showed that Kir2.1 protein was reduced in ventricular cardiomyocytes after myocardial infarction, and BMSCs transplantation can reverse these changes. * p<0.05 vs control, # p<0.05 vs MI.
